# Evaluation of CTB-sLip for Targeting Lung Metastasis of Colorectal Cancer

**DOI:** 10.3390/pharmaceutics14040868

**Published:** 2022-04-15

**Authors:** Xiaoying Zhang, Wenjing Tang, Haoyu Wen, Ercan Wu, Tianhao Ding, Jie Gu, Zhongwei Lv, Changyou Zhan

**Affiliations:** 1 Department of Nuclear Medicine, The Affiliated Shanghai Tenth People’s Hospital of Nanjing Medical University, Shanghai 200072, China; yingzx2002@126.com; 2 Center of Medical Research and Innovation, Shanghai Pudong Hospital & Department of Pharmacology, School of Basic Medical Sciences, Fudan University, Shanghai 201399, China; 16111030025@fudan.edu.cn (W.T.); 18111010017@fudan.edu.cn (T.D.); 3 Department of Thoracic Surgery, Zhongshan Hospital, Fudan University, Shanghai 200032, China; 20211210051@fudan.edu.cn (H.W.); gu.jie3@zs-hospital.sh.cn (J.G.); 4 School of Pharmacy, Fudan University & Key Laboratory of Smart Drug Delivery (Fudan University), Ministry of Education, Shanghai 201203, China; 20211030020@fudan.edu.cn

**Keywords:** lung metastasis, targeting, colorectal cancer, liposome, immunogenicity

## Abstract

Lung metastasis of colorectal cancer is common in the clinic; however, precise targeting for the diagnosis and therapy purposes of those lung metastases remains challenging. Herein, cholera toxin subunit b (CTB) protein was chemically conjugated on the surface of PEGylated liposomes (CTB-sLip). Both human-derived colorectal cancer cell lines, HCT116 and HT-29, demonstrated high binding affinity and cellular uptake with CTB-sLip. In vivo, CTB-sLip exhibited elevated targeting capability to the lung metastasis of colorectal cancer in the model nude mice in comparison to PEGylated liposomes (sLip) without CTB modification. CTB conjugation induced ignorable effects on the interaction between liposomes and plasma proteins but significantly enhanced the uptake of liposomes by numerous blood cells and splenic cells, leading to relatively rapid blood clearance in BALB/c mice. Even though repeated injections of CTB-sLip induced the production of anti-CTB antibodies, our results suggested CTB-sLip as promising nanocarriers for the diagnosis of lung metastasis of colorectal cancer.

## 1. Introduction

Colorectal cancer has become an increasing burden in public health, firmly ranking in the top three of both the morbidity and the cause of death from malignant tumors [1]. Even worse, remote spreading of colorectal cancer is of high occurrence, which accelerates the pathological progression and exacerbates the difficulty of clinical treatments. Lung metastasis is among the most common in colorectal cancer patients even after surgical resection [2], readily leading to rapid recurrence and disease progression. However, the early stage of lung metastasis of colorectal cancer remains challenging to track even using positron emission tomography [3]. Targeting the lung metastasis of colorectal cancer is of crucial importance for the diagnosis and therapy purposes [4].

There are numerous ligands being developed to target colorectal cancer cells by recognizing the corresponding receptors or antigens, including small molecules [5], peptides [6], antibodies [7], and aptamers [8]. Such targeting ligands are conjugated with nanocarriers for the delivery of imaging probes [9], therapeutic agents [10], or even both. Unfortunately, none of them have been successfully approved for the clinical use. There are multiple biological barriers hampering the efficient delivery of those targeted delivery systems [11]. We [12,13] and other groups [14,15] previously reported that ligand modification could severely alter the bio-nano surface properties, thus affecting the formation of protein corona and enhancing the mononuclear phagocyte system recognition. Conversely, the biological media that the targeted delivery systems encounter during in vivo circulation can also destroy the ligands to compromise the bioactivity [16,17], leading to the loss of targeting capability.

GM1 ganglioside is expressed on the membrane of various types of cells [18], acting as the receptor for cholera toxin by strongly binding with the subunit b (CTB) [19]. In our previous study [20,21], the CTB protein was conjugated on the surface of poly (lactic-co-glycolic acid) (PLGA) nanoparticles to penetrate the blood–brain barrier and to target glioma cells and neovasculature by recognizing GM1. The CTB protein not only preserved bioactivity in the presence of mouse serum but also exhibited nonsignificant impacts on the absorption of plasma proteins after conjugation on the surface of PLGA nanoparticles. These results suggested that the CTB protein may be an appropriate ligand to efficiently circumvent the biological barriers in blood circulation by mitigating the reciprocal influences between delivery systems and biological media.

The CTB protein was found to demonstrate high binding with intestinal cells in the human histological sections [22,23], suggestive of the potential of the CTB protein for targeting colorectal cancer. Liposomes are a class of commonly used nanocarriers in both basic research [24,25] and the clinic [26], representing a class of versatile tools for the delivery of imaging probes and therapeutic agents [27]. In the present study, the CTB protein was chemically conjugated on the surface of PEGylated liposomes (CTB-sLip). We investigated the targeting capability of CTB-sLip to colorectal cancer cells in vitro and lung metastasis of colorectal cancer in vivo. In particular, the effects of serum were emphasized on the bioactivity of the CTB protein, as well as the immunogenicity and in vivo fate of CTB-sLip.

## 2. Materials and Methods

### 2.1. Animals and Cells

Adult male BALB/c mice and nude mice were purchased from Shanghai Laboratory Animal Research Center (Shanghai, China) and raised under Specific Pathogen Free (SPF) conditions. HCT116 and HT-29 cell lines were purchased from Shanghai Institute of Cell Biology. Cells were cultured with McCoy’5A medium containing 10% fetal bovine serum (FBS) at 37 °C with 5% CO_2_.

### 2.2. Materials

Cholesterol, mPEG_2000_-DSPE, Mal-PEG_3500_-DSPE, and HSPC were acquired from Shanghai A.V.T. Pharmaceutical company (Shanghai, China). DiI (1,1′-Dioctadecyl-3,3,3′,3′-tetramethylindocarbo-cyanine perchlorate), 2-iminothiolane hydrochloride (Traut’s Reagent), and Sephadex^®^ G50 were obtained from Sigma (St. Louis, MO, USA). Doxorubicin HCl/Adriamycin (MB1087-S), DiO dye (MB4239), and DiR dye (MB12482) were bought from Meilunbio Co. Ltd. (Dalian, China). HisSep Ni-NTA Agarose Resin 6FF (20503ES50) and Ampicillin Sodium (60203ES10) were acquired from YEASEN Biotech Co., Ltd. (Shanghai, China). One-Step™ ABTS Substrate Solution (37615), Zeba™ Spin Desalting Columns (7 kDa MWCO) and a Pierce TM BCA Protein Assay Kit (23225) were obtained from Thermo Fisher Scientific (Rockford, IL, USA). TMB Substrate Solution for ELISA was obtained from Beyotime Biotech Co. Ltd. (Shanghai, China). Anti-His tag antibody [M2] (HRP) (ab9108), anti-mouse serum albumin (ab19194), and donkey polyclonal secondary antibody to Rabbit IgG-H&L (Alexa Fluor^®^ 647) (ab150075) were obtained from Abcam (Cambridge, MA, USA). Mini-PROTEAN^®^ TGXTM Precast Protein Gel 4–20% (4561093) was purchased from Bio-Rad Laboratories Co., Ltd. (Shanghai, China). Polymyxin B sulfate (A610318), IPTG (Isopropyl-beta-D-thiogalactopyranoside), and MOPS (4-Morpholinepropanesulfonic acid) were purchased from Sangon Biotech Co., Ltd. (Shanghai, China). Tryptone (LP0042) and yeast extract (LP0021) were obtained from Oxid Co., Ltd. (Hants, UK). Ganglioside GM1 was purchased from Avanti Co., Ltd. (Birmingham, UK). McCoy’5A medium (L630KJ) was obtained from Basal Media Co., Ltd. (Shanghai, China), and FBS was obtained from Cytiva Co., Ltd. (Marlborough, MA, USA). APC anti-mouse CD3 antibody (100235), Brilliant Violet 421^TM^ anti-mouse CD19 antibody (115537), APC anti-mouse/human CD11b antibody (101211), Brilliant Violet 421™ anti-mouse Ly-6G antibody (127627), APC anti-mouse CD146 antibody (134711), Brilliant Violet 421™ anti-mouse F4/80 antibody (123131), anti-mouse CD11c recombinant antibody (161102), and Brilliant Violet 421™ anti-human CD326 (EpCAM) Antibody (369821) were purchased from BioLegend. Co., Ltd. (San Diego, CA, USA).

### 2.3. Expression and Characterization of the CTB Protein

The plasmid was successfully constructed and cloned into PET-28a (kanamycin resistance) containing a C-terminal His (6×) tag. The *E. Coli* cells were amplified at 37 °C and induced at 15 °C by IPTG (1 mM) for 12 h. The crude protein was obtained by centrifugation at 6000× *g* for 10 min and dissolved in buffer containing 50 mM Tris, 500 mM NaCl, 2 M urine, and 10 mM 2-hydroxy-1-ethanethiol. The crucial protein was folded by dialysis for 48 h and subsequently loaded in a Ni-NTA column according to manufacturer’s instructions (Yisen Co. Ltd., Shanghai, China). The CTB protein was collected with elution buffer (250 mM imidazole in PBS) and ultrafiltrated at 8000× *g* with PBS three times until complete replacement of the storing buffer by PBS. The gradient (4–20%) polyacrylamide SDS-PAGE gel and Fast sliver staining kit were used to characterize the purity and molecular weight of the CTB protein.

ELISA was used to determine the binding activity of CTB to GM1. GM1 was coated on 96-well microplates with 1 μg per well at 4 °C overnight. After blockade with 5% BSA (in PBS), serial dilutions of CTB in 0.1% BSA were added and incubated at 37 °C for 1 h. HRP conjugated anti-His antibody was used to detect CTB at 405 nm (ABTS).

### 2.4. Preparation and Characterization of Liposomes

Plain PEGylated liposomes (sLip) were prepared by the lipid-film hydration method. HSPC, cholesterol, and mPEG_2000_-DSPE (52:43:5 in molar ratio) were dissolved in chloroform and formed a thin film through vacuum evaporation. Any residual chloroform was removed overnight under vacuum. The film was hydrated with saline and extruded through polycarbonate membranes (400 nm, 200 nm, and 100 nm) at 60 °C. The DiI-, DiR-, and DiO-labelled sLip (sLip/DiI, sLip/DiR, and sLip/DiO) were prepared according to the same procedure as above except by adding 100 μg mL^−1^ DiI, DiR, and DiO, respectively. The 5-FAM-loaded sLip (sLip/FAM) were prepared using the same procedure as that used for plain sLip, except using 5-FAM solution (2 mg mL^−1^ in deionized water) to hydrate the film. A Sephadex-G50 column was used to remove the unloaded fluorescence dye. Maleimide functionalized sLip were prepared by adding 1% mol Mal-PEG_3500_-DSPE before vacuum evaporation.

CTB (775 μg, 14 nmol) was dissolved in 1.5 mL PBS (containing 5 mM EDTA, pH adjusted to 8.0), and 9.7 μL Traut’s Reagent (14.5 mM in PBS) was added. The mixture was reacted for 45 min at room temperature. A desalting column was used to remove un-reacted Traut’s Reagent, and the thiolated CTB was collected by centrifugation. Mal-sLip (1 mL, with 10 μmol HSPC) were mixed with thiolated CTB (1–4 nmol, resulted in a molar ratio of 0.1–0.4‰ CTB to HSPC). The mixture was reacted at room temperature for 3 h. The reaction solution was collected, and CTB-sLip were purified using a Sephadex-G50 column. The size and zeta potential of liposomes (50 times dilution with deionized water) were measured by a Zetasizer Nano ZS (Malvern Instruments, Malvern, UK).

### 2.5. Characterization of Liposome Stability in Mouse Serum

sLip/DiI and CTB-sLip/DiI were incubated with the same volume of BALB/c serum for 24 h at room temperature, and PBS was set as a control. Liposomes were diluted 1000 times and detected by Nanoparticle Tracking Analysis (NTA 3.4 Build 3.4.003).

### 2.6. Binding Activity of CTB-sLip with GM1 In Vitro

GM1 or BSA was coated in 96-well microplates with 1 μg per well. After blockade with 5% BSA in PBS, serial dilutions of CTB-sLip/DiI were added and reacted at 37 °C for 1 h; sLip/DiI were set as the controls. DiI was quantified by a microplate reader at Ex/Em = 540/580 nm. To evaluate the effect of serum on GM1 binding, CTB-sLip were pre-incubated with BALB/c serum or PBS for 24 h. Serial dilutions were added and incubated with GM1-immoblized wells at 37 °C for 1 h. HRP conjugated anti-His antibody was used to detect CTB at 450 nm (TMB).

### 2.7. Protein Corona Separation and Characterization

CTB-sLip with different CTB modification degrees or sLip were incubated with the same volume of BALB/c plasma at 37 °C for 1 h. After centrifugation at 14,000× *g* for 30 min, the pellet was rinsed with cold PBS twice. The pellet was boiled in a mixture containing SDS-PAGE 5 × sample buffer (5 μL), PBS (20 μL) and β-mercaptethanol (2 μL) at 95 °C for 5 min. Electrophoresis was performed using gradient polyacrylamide gel, which was stained with a Fast Silver Stain Kit.

### 2.8. Pharmacokinetic Profile of CTB-sLip/DiI

Male BALB/c aged 4–5 weeks were intravenously injected with CTB-sLip or sLip (50 mg kg^−1^ lipid), and blood was sampled at 10 min, 30 min, 1 h, 2 h, 4 h, 8 h, 12 h, and 24 h. The fluorescence of DiI in plasma was detected by a microplate reader at 540/580 nm.

### 2.9. Cellular Uptake and Binding of CTB-sLip/FAM In Vitro

HCT116 and HT-29 cells were cultured with McCoy’5A medium (10% FBS) at 5% CO_2_. Cells were seeded into 12-well plates and incubated with CTB-sLip/FAM or sLip/FAM (5 μM of FAM) in 0.1% BSA or 10% BALB/c serum for 2 h at 37 °C. Cells were harvested and analyzed by flow cytometry. To assess the binding ability of CTB-sLip/FAM or sLip/FAM with cells, liposomes (5 μM of FAM) in 0.1% BSA or 10% BALB/c serum were incubated with HCT116 and HT-29 cells for 2 h at 4 °C.

### 2.10. Uptake of CTB-FITC to Colorectal Cancer Cell Lines

HCT116 and HT-29 cells were cultured and seeded as aforementioned. The FITC-labeled CTB protein (CTB-FITC, 1 μM) was added and incubated with cells in the presence of BSA or BALB/c serum for 2 h at 37 °C. FITC labeled BSA (BSA-FITC) was set as the control. Flow cytometry was used to evaluate the uptake ability of the CTB protein in colorectal cancer cells.

### 2.11. Uptake of Liposomes by Blood Cells of BALB/c Mice In Vivo

To investigate the cellular uptake of CTB-sLip in peripheral blood cells, male BALB/c mice aged 4–5 weeks were intravenously injected with CTB-sLip/DiO (50 mg kg^−1^ lipid, *n* = 3), and sLip/DiO were set as the control. Peripheral whole blood was sampled at 2 h or 24 h after injection and collected into PBS with 10 mM EDTA. Red blood cells were then lysed twice with ACK Lysis Buffer. Fluorescent antibodies, including anti-mouse CD3/APC for T cells, anti-mouse CD19/BV421 for B cells, anti-mouse CD11b/APC, and anti-mouse Ly6G/BV421 for monocytes and neutrophils, were incubated with white blood cells at 4 °C for 30 min. The fluorescence of internalized DiO-labeled liposomes was evaluated by flow cytometry.

### 2.12. Cellular Uptake of Liposomes in Liver and Splenic Cells In Vivo

BALB/c mice were intravenously injected with CTB-sLip/DiO (50 mg kg^−1^ lipid, *n* = 3); sLip/DiO were the control group. Mice were sacrificed at 2 h or 24 h, and the corresponding livers and spleens were dissected after perfusion with HBSS via postcava. The livers were gently mashed and sieved through a 70 μm cell strainer. Hepatocytes were collected by centrifugation (4 °C, 50× *g*, 3 min), and the supernatant containing nonparenchymal cells was collected and centrifuged at 650× *g* for 7 min. Kupffer cells and liver sinusoidal endothelial cells (LSECs) were enriched in the middle layer by Percoll gradient centrifugation (25%/50%). Hepatocytes were fixed in 4% PFA and permeabilized with 0.5% Triton-X 100. Collected cells were strained with anti-mouse albumin and Alexa Fluor 647 donkey anti-Rabbit IgG antibody. The enriched nonparenchymal cells were strained with anti-mouse F4_80/BV421 and anti-mouse CD146/APC to mark Kupffer cells and liver sinusoidal endothelial cells (LSEC).

The dissected spleens were sieved through a 70 μm cell strainer, and spleen cell suspensions were incubated with anti-mouse CD3/APC, anti-mouse CD19/BV421, anti-mouse F4_80/BV421, and anti-mouse CD11c antibody at 4 °C for 30 min to mark T cells, B cells, Macrophages (Mϕ), and DC cells. The fluorescence of internalized liposomes was evaluated by flow cytometry (Agilent Technologies, Novocyte3000, Santa Clara, CA, USA).

### 2.13. Distribution of Liposomes in BALB/c Nude Mice with Lung Metastasis of Colorectal Cancer

Male BALB/c nude mice aged 6–8 weeks were injected with 2 × 10^6^ HCT116 or HT-29 cells per mice in the left lung, and distribution of liposomes was determined after 9–14 days. CTB-sLip/DiR or sLip/DiR (50 mg kg^−1^ lipid) were intravenously injected, and organs were dissected at 24 h after administration. Opti-Scan Viewn Vivo superior optics (vie-works, for HT-29 cells animal model) and Visque InVivio Elite (for HCT116 cells animal model) were applied to quantify the fluorescence intensity of organs. DiO-labeled liposomes (50 mg kg^−1^ lipid per kg body weight of mice) were applied to evaluate cellular distribution in vivo. Lungs were dissected at 24 h after injection and digested with IV collagenase. The tumor cells were labeled with BV421 conjugated anti-CD236, and DiO in tumor cells was analyzed by flow cytometry (Agilent Technologies, Novocyte3000, Santa Clara, CA, USA).

### 2.14. Immunogenicity of Liposomes

Male BALB/c nude mice weighing ~20 g were intravenously injected through the tail vein with CTB-sLip or sLip (50 mg kg^−1^) per week for 21 days, and the corresponding serum of each group was sampled. sLip or CTB-sLip (20 μg lipid per well) were coated in 96-well microplates, and serial dilutions of corresponding serum were added and incubated at 37 °C for 1 h. HRP-conjugated anti-mouse IgM and IgG antibody were utilized to detect anti-PEG IgG/IgM and anti-CTB IgG/IgM in serum, respectively.

### 2.15. Statistical Analysis

Data are presented as the means ± standard deviations (SD) and were analyzed by GraphPad Prism software 8.0.1 (GraphPad Software, San Diego, CA, USA). *p* < 0.05 was considered statistically significant (ns: *p* > 0.05, * *p* < 0.05, ** *p* < 0.01, *** *p* < 0.001).

## 3. Results and Discussion

### 3.1. Preparation and Characterization of CTB-Conjugated Liposomes (CTB-sLip)

**Figure 1:**
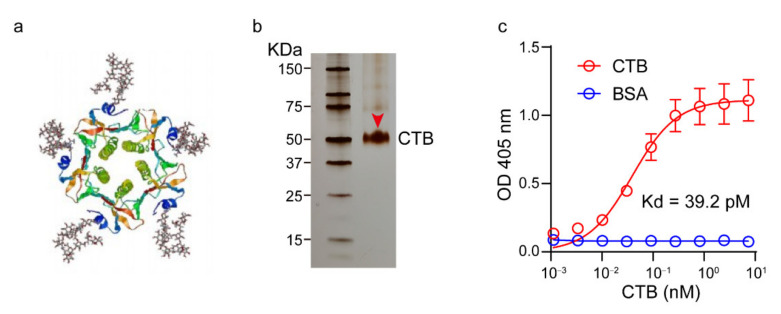
Expression and characterization of the CTB protein. (**a**) Schematically illustration of the structure of CTB. (**b**) Characterization of the expressed and refolded CTB by SDS-PAGE. (**c**) Binding activity of the expressed CTB to GM1; BSA was set as the control. The K_d_ value was calculated using GraphPad Prism 8.0.1. Data are means ± SDs, *n* = 3.

The CTB protein is a homopentamer that binds GM1 in each monomer (Figure 1a). A His (6×) tag was constructed in the CTB protein molecule for purification. After expression via the *E. coli* system and protein refolding (see Methods), the CTB protein was purified by the Ni-NTA column with good purity and correct molecular weight according to the SDS-PAGE silver staining results (Figure 1b). The purified CTB protein was quantified by the BCA method, demonstrating an expression yield of 10–15 mg L^−1^ in the present study. The expressed CTB protein was further ascertained by measuring its binding affinity with GM1 using the ELISA method (see Methods). As shown in Figure 1c, the expressed CTB protein demonstrated high binding affinity with GM1, registering an equilibrium dissociation constant (K_d_) of 39.2 pM. In contrast, the negative control protein, BSA, exhibited no obvious binding with GM1.

**Figure 2:**
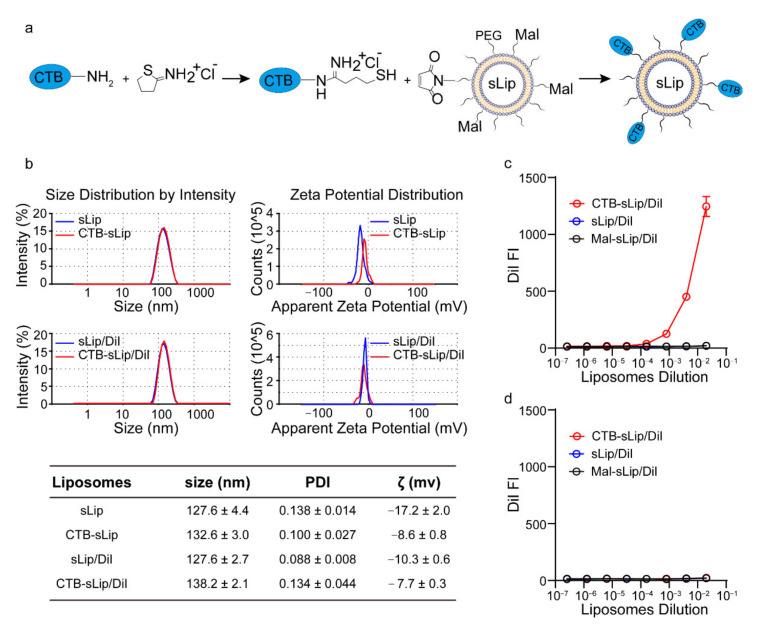
Preparation and characterization of CTB-conjugated PEGylated liposomes (CTB-sLip). (**a**) Schematic illustration of the conjugation method of CTB to the maleimide group grafted on the surface of PEGylated liposomes. (**b**) Size and zeta potential characterization of the prepared CTB-sLip and fluorescence dye DiI-loaded CTB-sLip. The binding activities of CTB-sLip/DiI to GM1 coated (**c**) and BSA coated (**d**) were performed by ELISA; sLip/DiI and Mal-sLip/DiI were set as the controls. Data are means ± SDs, *n* = 3.

To chemically conjugate the CTB protein on the surface of PEGylated liposomes, the CTB protein was thiolated using Traut’s Reagent (see Methods) and reacted with the maleimide group of PEGylated liposomes (Figure 2a). As shown in Figure 2b, all populations of liposomes demonstrated a mean diameter ~130 nm with narrow size distributions. The conjugation of CTB slightly increased particle sizes and zeta potentials. The binding of CTB-sLip with GM1 was measured in the 96-well ELISA plates by reading the fluorescence of DiI encapsulated in liposomes (Figure 2c). CTB-sLip demonstrated increasing fluorescence intensity of the encapsulated DiI in a concentration-dependent manner, indicating preservation of CTB bioactivity after chemical conjugation with PEGylated liposomes. In contrast, sLip and Mal-sLip (without CTB modification) displayed no binding with GM1. Meanwhile, the nonspecific binding of all populations of liposomes was excluded by measuring the interactions of liposomes with BSA-coated plates (Figure 2d).

### 3.2. Effects of Serum on the Targeting Capability of CTB-sLip

**Figure 3:**
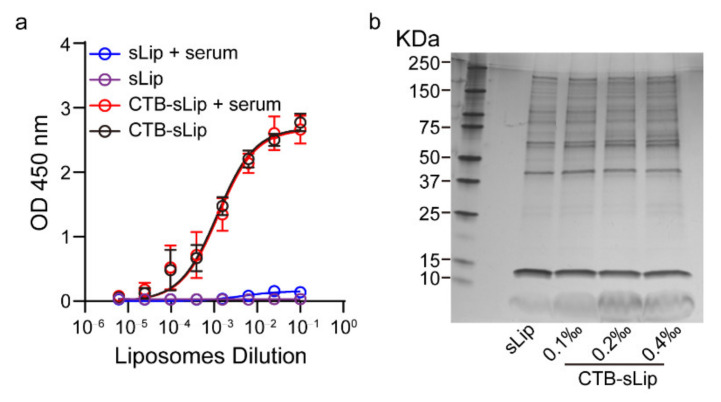
Effects of BALB/c plasma proteins on the bioactivity of CTB-sLip. (**a**) Affinity characterization of CTB-sLip (pre-incubation with BALB/c serum and PBS for 24 h) to GM1 based on ELISA; sLip were set as a control. Data are means ± SDs, *n* = 3. (**b**) Evaluation of protein corona of CTB-sLip (with 0.1‰, 0.2‰ or 0.4‰ molar ratio CTB modification) by SDS-PAGE.

The effects of mouse serum on CTB-sLip were investigated in vitro. After incubation with BALB/c serum for 24 h, liposomal size and distribution were measured using Nanoparticle Tracking Analysis (NTA). As shown in Figure S1, all particles in various media displayed a major size distribution ranging from 50 nm to 300 nm. Pre-incubation of CTB-sLip or sLip with BALB/c serum did not significantly alter the size distribution. The effect of serum on CTB bioactivity was also determined. In comparison to PBS (Figure 3a), pre-incubation with BALB/c serum only slightly affected the CTB binding to GM1. The formed protein coronas on the surface of CTB-sLip and sLip were separated, and their compositions were analyzed using SDS-PAGE. As shown in Figure 3b, the CTB protein at different modification degrees did not induce obvious differences in the composition of the formed protein corona in comparison to sLip. These results are consistent with our previous report [20], where the CTB protein on the surface of the polymeric nanoparticles preserved bioactivity with GM1 after interaction with serum.

**Figure 4:**
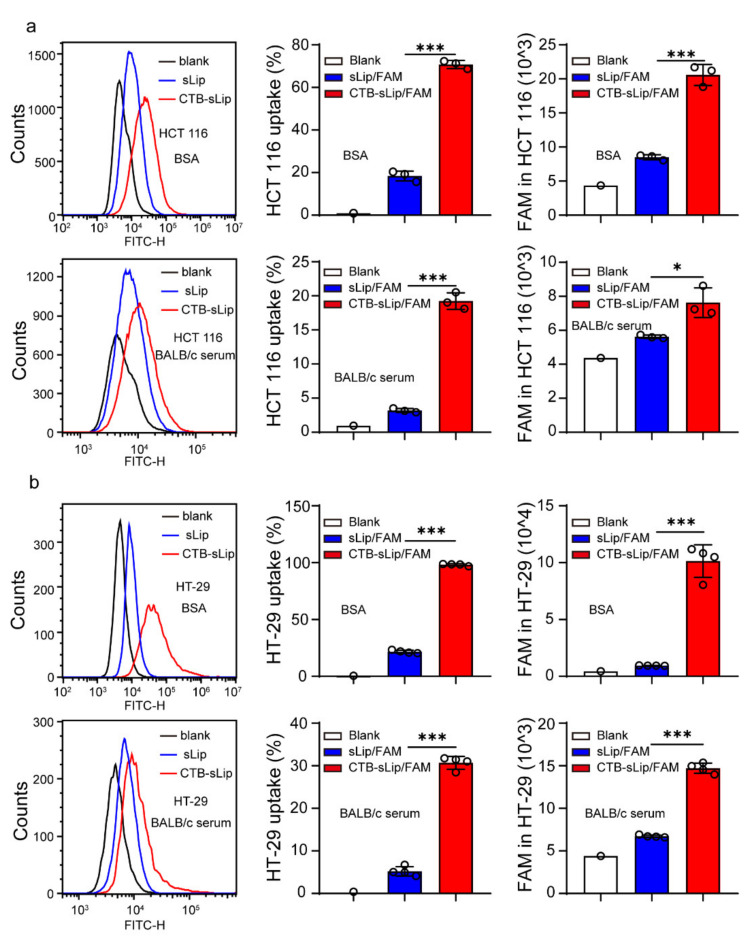
Effect of BALB/c serum on the targeting capability of CTB-sLip to human-derived colorectal cancer cells. CTB-sLip/FAM (5 μM 5-FAM) were prepared to study the in vitro uptake by HCT116 cell ((**a**), *n* = 3) and HT-29 cell ((**b**), *n* = 4) at 37 °C after 2 h incubation, in 0.1% BSA and 10% BALB/c serum, respectively. Data are means ± SDs. * *p* < 0.05 and *** *p* < 0.001 based on one-way ANOVA with Prism.8.0.1.

To study the targeting capability of CTB-sLip, two human-derived colorectal cancer cell lines, HCT116 and HT-29, were cultured. As shown in Figure S2, both cell lines demonstrated high uptake of CTB, suggesting GM1 expression on the cell membrane. The binding of CTB-sLip with cells was studied at 4 °C. After 2 h incubation with FAM-encapsulated CTB-sLip or sLip, the FAM fluorescence-positive cells were counted using a flow cytometer. As shown in Figures S3 and S4, both HCT116 and HT-29 cells demonstrated significantly higher binding with CTB-sLip than with sLip, suggesting the targeting capability of CTB-sLip to human-derived colorectal cancer cells. The presence of BALB/c serum dramatically decreased the binding of all populations of liposomes with cancer cells. However, CTB-sLip still exhibited significant enhancement of cell binding compared with sLip, which is consistent with the results shown in Figure 3. Cellular uptake of liposomes by cancer cells was conducted at 37 °C (Figure 4). As expected, CTB demonstrated high targeting capability to both HCT116 and HT-29 cells.

### 3.3. Pharmacokinetic Profiles of CTB-sLip

**Figure 5:**
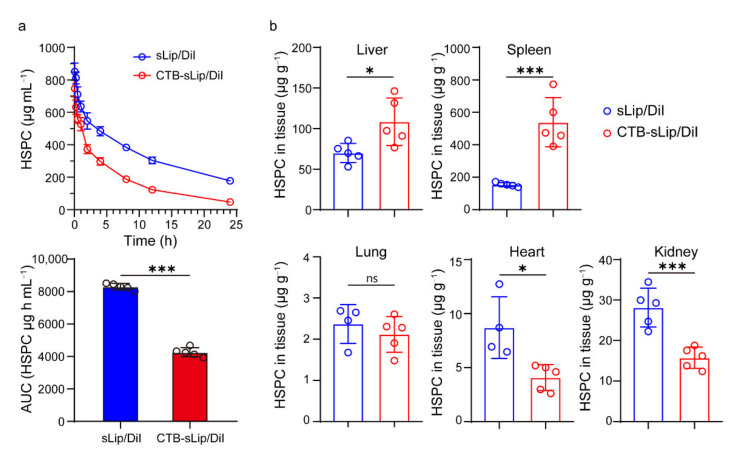
Pharmacokinetic profiles (**a**) and biodistribution (**b**) of sLip/DiI and CTB-sLip/DiI in major organs of BALB/c mice over 24 h after intravenous injection with 50 mg kg^−1^ lipids. Data are means ± SDs, *n* = 5. * *p* < 0.05, *** *p* < 0.001; ns indicates non-significant based on Student’s *t*-tests.

To study the in vivo fate of liposomes, the pharmacokinetic profiles and biodistribution of both CTB-sLip and sLip were investigated in BALB/c mice. Liposomes at a dose of 50 mg kg^−1^ (lipid to mouse body weight) were injected into the tail vein, and blood was sampled at the predetermined time points. As shown in Figure 5a, CTB-sLip demonstrated a significant decrease of the area under the curve (AUC) in the plasma concentration–time curve, suggesting relatively rapid clearance of CTB-sLip in mice. At the last time point (24 h after injection), the biodistribution of liposomes in the main organs was measured (Figure 5b). Most liposomes were, as expected, found in the liver and spleen, and CTB-sLip still demonstrated higher distribution in both organs in comparison to sLip.

**Figure 6:**
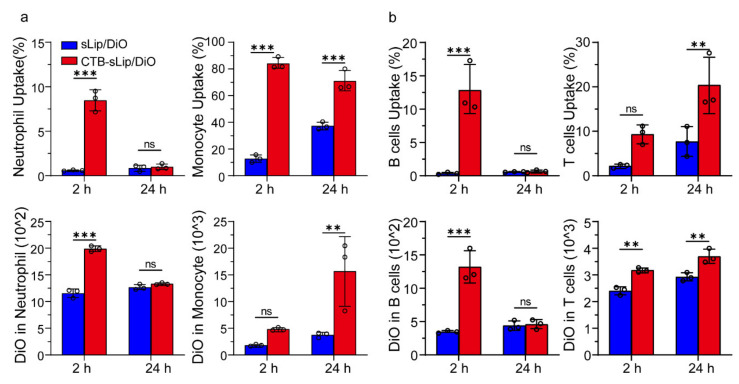
Uptake of CTB-sLip by blood white cells of BALB/c mice in vivo. CTB-sLip/DiO were injected with 50 mg kg^−1^ lipids. Uptake by neutrophils and monocytes (**a**), and T cells and B cells (**b**) in peripheral blood at 2 h and 24 h after injection was determined by Flow Cytometry. Data are means ± SDs (*n* = 3). ** *p* < 0.01, *** *p* < 0.001; ns indicates nonsignificant based on grouped two-way ANOVAs.

To further understand the interaction of CTB-sLip with the body, the uptake of liposomes by blood cells was measured. At 2 h and 24 h after injection of DiO-labeled liposomes, blood cells were collected and labeled with respective specific antibodies. The uptake of liposomes was determined using a flow cytometer. As shown in Figure 6, CTB-sLip demonstrated higher uptake of neutrophils, monocytes, B cells, and T cells to some extent, suggesting extensive binding of CTB-sLip with cells in vivo.

**Figure 7:**
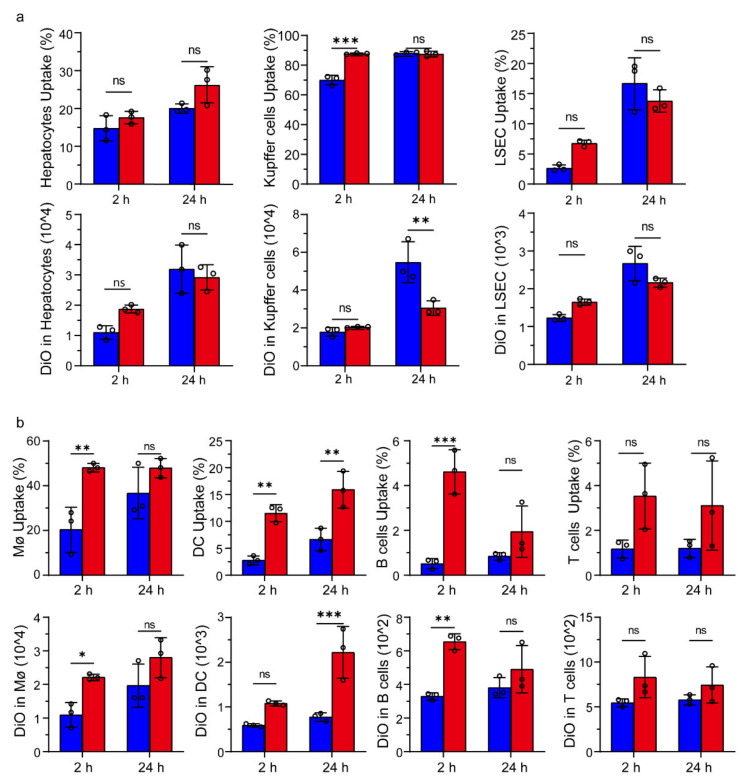
Uptake of CTB-sLip/DiO by hepatic cells and splenic cells. (**a**) Kupffer’s cells and liver sinusoidal endothelial cells (LSECs) were marked with F4/80 and CD146, respectively. (**b**) Splenic macrophages (MΦ), DC cells, splenic B cells, and T cells were marked with F4/80, CD11c, CD19, and CD3, respectively. Data are means ± SDs (*n* = 3). * *p* < 0.05, ** *p* < 0.01, *** *p* < 0.001; ns represents nonsignificant based on grouped two-way ANOVAs.

The biodistribution of liposomes in the liver and spleen of BALB/c mice was also carefully studied. The hepatocytes, Kupffer’s cells, and liver sinusoidal endothelial cells (LSECs) were separated according to the previously reported protocol with minor modification [28] (see Methods) and labeled with respective markers or antibodies. It was interesting that only CTB-sLip demonstrated significant enhancement (88% vs. 70%) of uptake by Kupffer’s cells in comparison to sLip (Figure 7a), while the other cells did not exhibit significant uptake of CTB-sLip at both time points. In contrast, CTB-sLip displayed significant enhancement of uptake by splenic cells, including splenic microphages, dendritic cells, and B cells (Figure 7b), which is consistent with the data shown in Figure 5b. At 24 h after injection, CTB-sLip exhibited higher accumulation in the spleen than sLip. These data also indicated that interaction of CTB-sLip with blood cells and splenic cells may majorly contribute to the relatively rapid clearance of CTB-sLip (Figure 5a).

### 3.4. Lung Metastasis Targeting of Colorectal Cancer by CTB-sLip

**Figure 8:**
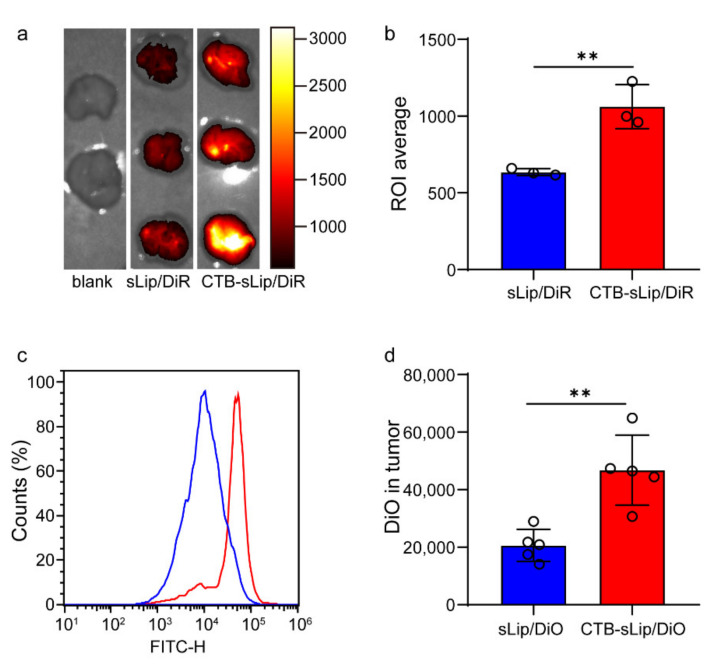
Targeting capability of CTB-sLip to lung metastasis of colorectal cancer in vivo. Male BALB/c nude mice planted with HT-29 cells in left lungs were injected with CTB-sLip/DiR ((**a**,**b**), *n* = 3) for in situ imaging and CTB-sLip/DiO ((**c**,**d**), *n* = 5) for cellular uptake study; sLip/DiR and sLip/DiO were set as the controls. Data are means ± SDs. ** *p* < 0.01 based on Student’s *t*-tests.

To evaluate the targeting capability of CTB-sLip to lung metastasis of colorectal cancer, male BALB/c nude mice were injected with 2 × 10^6^ HT-29 cells per mice in the left lung. At nine days after injection, fluorescent dye (DiO for cellular study and DiR for in situ imaging)-labeled liposomes (50 mg kg^−1^ lipid of mice body weight) were intravenously injected, and the left lung with implanted colorectal cancer cells was dissected for in situ imaging. As shown in Figure 8a,b, CTB-sLip demonstrated significant enhancement in the cancer tissues compared with sLip. The cells were further separated, and cellular uptake of liposomes was determined by a flow cytometer (Figure 8c,d). As shown in Figure S6, BALB/c nude mice with lung metastasis of HCT116 also demonstrated significantly higher distribution with CTB-sLip than with sLip at 24 h after injection, suggesting the targeting capability of CTB-sLip to human-derived colorectal cancer cells. Both CTB-sLip and sLip demonstrated higher uptake by cancer cells than the paracancerous lung tissues, which may be attributed to the enhanced permeability and retention effect. As for the cancer cells, CTB-sLip displayed significantly higher targeting capability than sLip, suggestive of the targeting capability of the former for lung metastasis of colorectal cancer.

### 3.5. Immunogenicity of CTB-sLip

**Figure 9:**
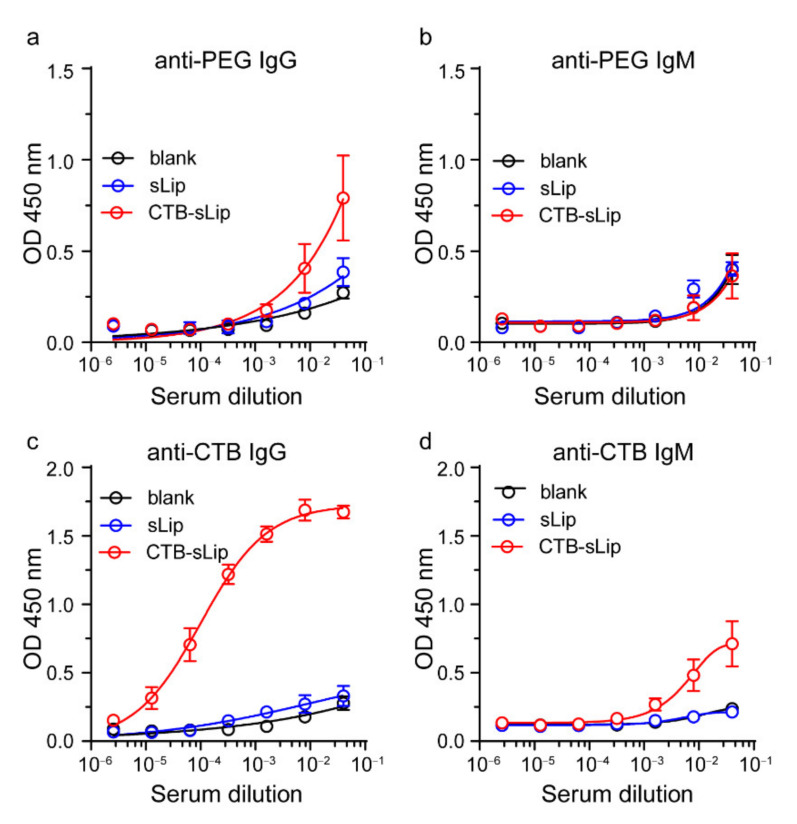
Immunogenicity of CTB-sLip in vivo. BALB/c mice were sequentially injected with sLip or CTB-sLip (50 mg kg^−1^ lipids) per week for 21 days. Anti-PEG IgG/M (**a**,**b**) and anti-CTB IgG/M (**c**,**d**) in serum on day 21 were detected by ELISA. Data are means ± SDs (*n* = 3).

Considering the high uptake of CTB-sLip by the splenic cells, immunogenicity was investigated by detecting the antibodies. Both CTB-sLip and sLip were intravenously injected via the tail vein weekly, and blood was sampled one week after the third injection. The anti-PEG and anti-CTB antibodies were measured using the ELISA methods (see Methods). As shown in Figure 9, sequential injections of CTB-sLip induced significant anti-CTB IgG and slight anti-CTB IgM antibodies. Meanwhile, CTB-sLip demonstrated slight enhancement of anti-PEG IgG antibody in comparison to sLip, with no significant generation of anti-PEG IgM antibody. These data suggested the immunogenicity of CTB-sLip, warning the repeated injection when CTB-sLip were exploited as the targeted delivery system.

## 4. Conclusions

Herein, the CTB protein was chemically conjugated on the surface of PEGylated liposomes, and their potential for targeting lung metastasis of colorectal cancer was evaluated. Chemical conjugation of CTB did not significantly alter the interaction between liposomes and plasma proteins. CTB-sLip demonstrated good stability in the presence of mouse serum, which may be attributed to the high stability of the homopentamer structure and low plasma protein binding on the surface of CTB-sLip. The presence of serum induced the decrease of binding with colorectal cancer cells to some extent, while CTB-sLip still retained targeting capability in comparison to sLip. As expected, CTB demonstrated high targeting capability to lung metastasis of colorectal cancer. The extensive interaction of CTB-sLip with blood cells and splenic cells may contribute to their relatively rapid blood clearance from BALB/c mice. Even though immunogenicity of CTB-sLip warned their applications when repeated treatments were indispensable, the present study provided a potential tool to achieve lung metastasis targeting of colorectal cancer for diagnostic purposes.

## Supplementary Materials

The following supporting information can be downloaded at: https://www.mdpi.com/article/10.3390/pharmaceutics14040868/s1, Figure S1: Characterization of liposomes stability in BALB/c serum in vitro. Figure S2: The uptake capability of CTB protein to HCT 116 and HT-29 cells in vitro. Figure S3: The binding capability of CTB-sLip to HCT 116 cells in vitro. Figure S4: The binding capability of CTB-sLip to HT-29 cells in vitro. Figure S5: Characterization of antibody strained cells by flow-cytometry. Figure S6: Targeting capability of CTB-sLip to lung metastasis of colorectal cancer in vivo.
