# Supplementary material for: Evaluation of CTB-sLip for Targeting Lung Metastasis of Colorectal Cancer
Source: Pharmaceutics. 2022 Apr 15;14(4):868. doi: 10.3390/pharmaceutics14040868 (PMC9032673; doi:10.3390/pharmaceutics14040868)
Supplement: Supplementary file 1 [file pharmaceutics-14-00868-s001.zip › pharmaceutics-1637268-supplementary.pdf]

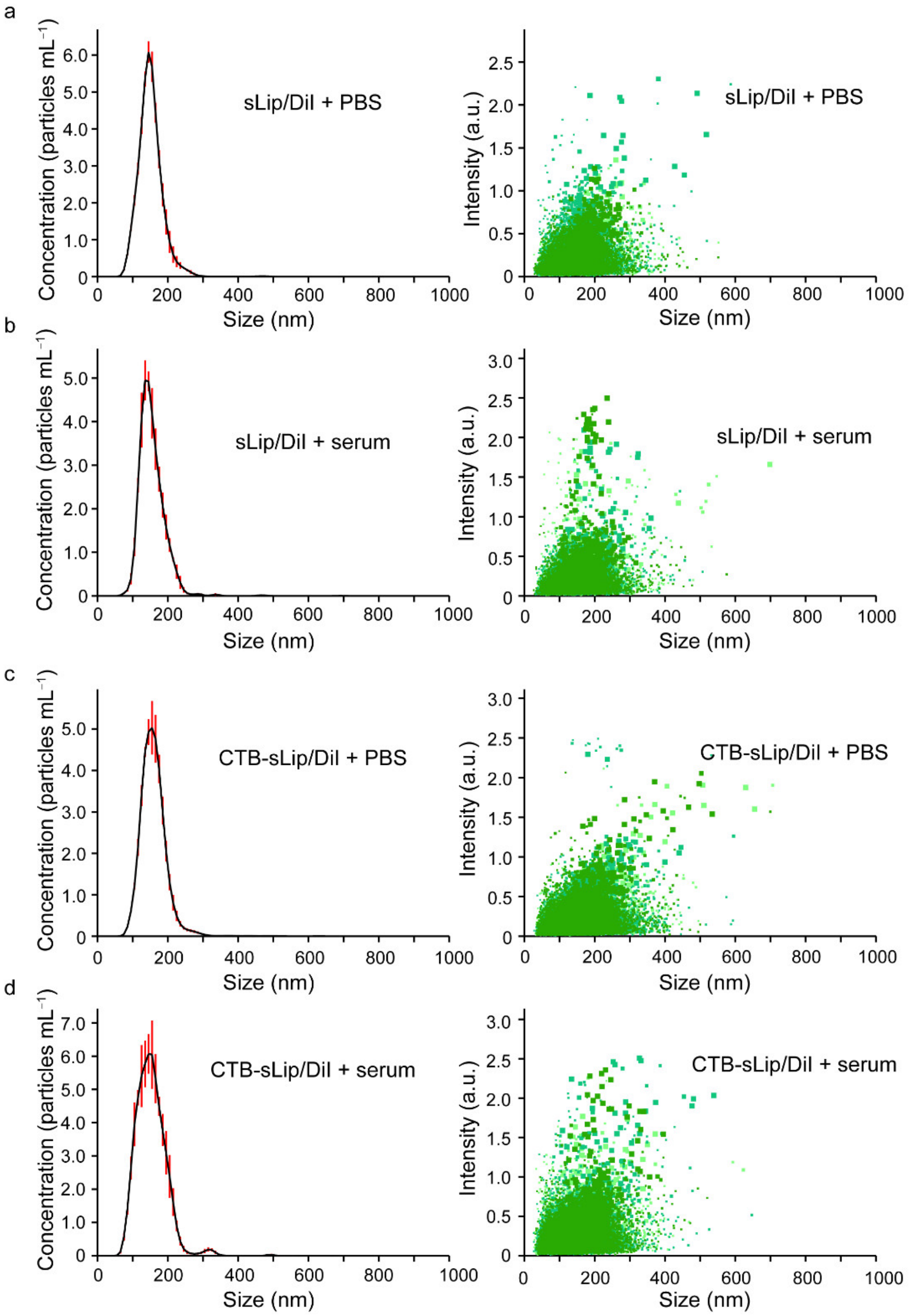

Supplementary Figure S1. Characterization of liposomes stability in BALB/c serum *in vitro*. sLip/DiI (a-b) or CTB-sLip/DiI (c-d) were incubated with PBS or BALB/c serum at room temperature for 24 h. The solution were 1000 times diluted and analyzed with NTA instrument.

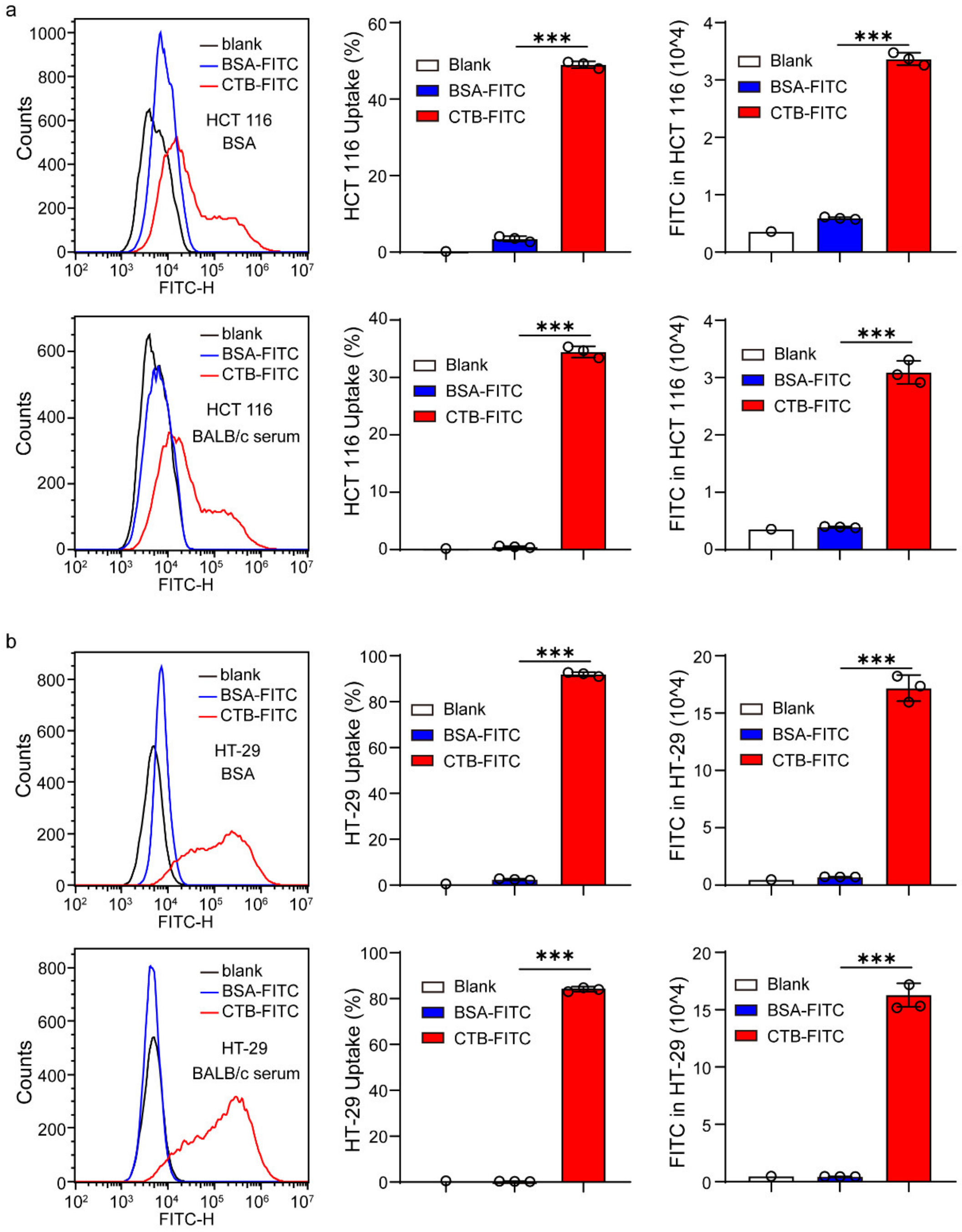

Supplementary Figure S2. The uptake capability of CTB protein to HCT 116 and HT-29 cells *in vitro*. HCT 116 cells (a) and HT-29 cells (b) were cultured and seeded with 3×10^5 cells in 12-well plates (n = 3). CTB-FITC was incubated with cells with the concentration of 1 μM at 37 ℃ for 2 h, and BSA-FITC was control. The blank for each cell line is the same when comparing the difference between BSA and BALB/c serum. Data are means ± SDs. *** $p < 0.001$ by one-way ANOVA analyses with Prism.8.0.1.

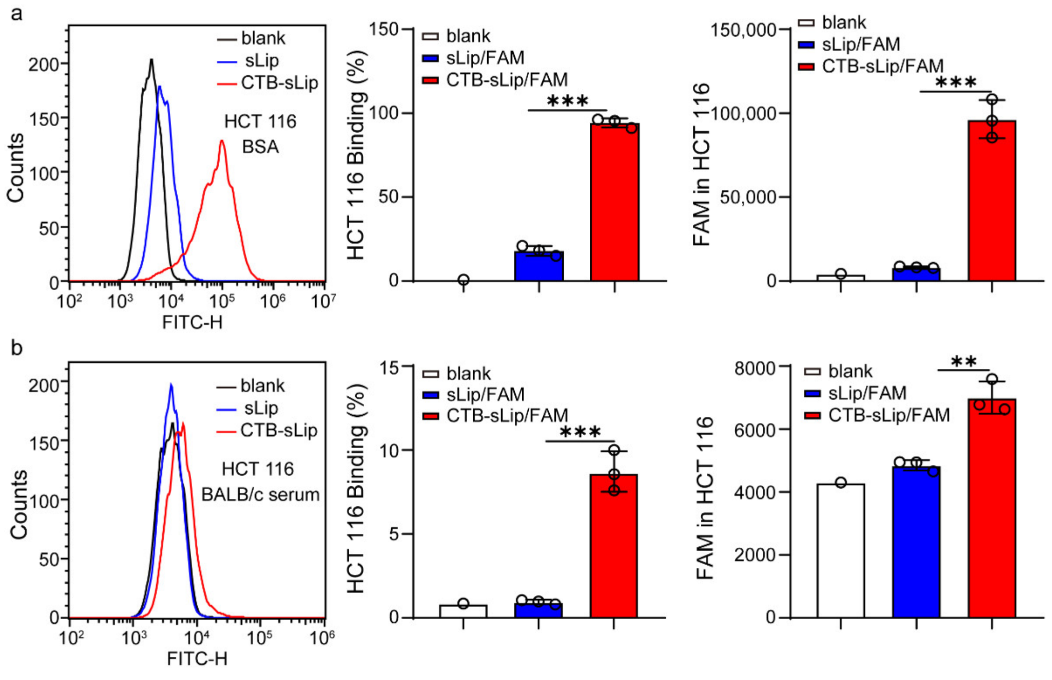

Supplementary Figure S3. The binding capability of CTB-sLip to HCT 116 cells *in vitro*. FAM loaded liposomes (5 µM FAM) were incubated with HCT 116 cells in 0.1 % BSA (a) and 10 % BALB/c serum (b) at 4 ℃ for 2 h (n =3). Data are means ± SDs. ** p < 0.01 and *** p < 0.001 by one-way ANOVA analyses with Prism.8.0.1.

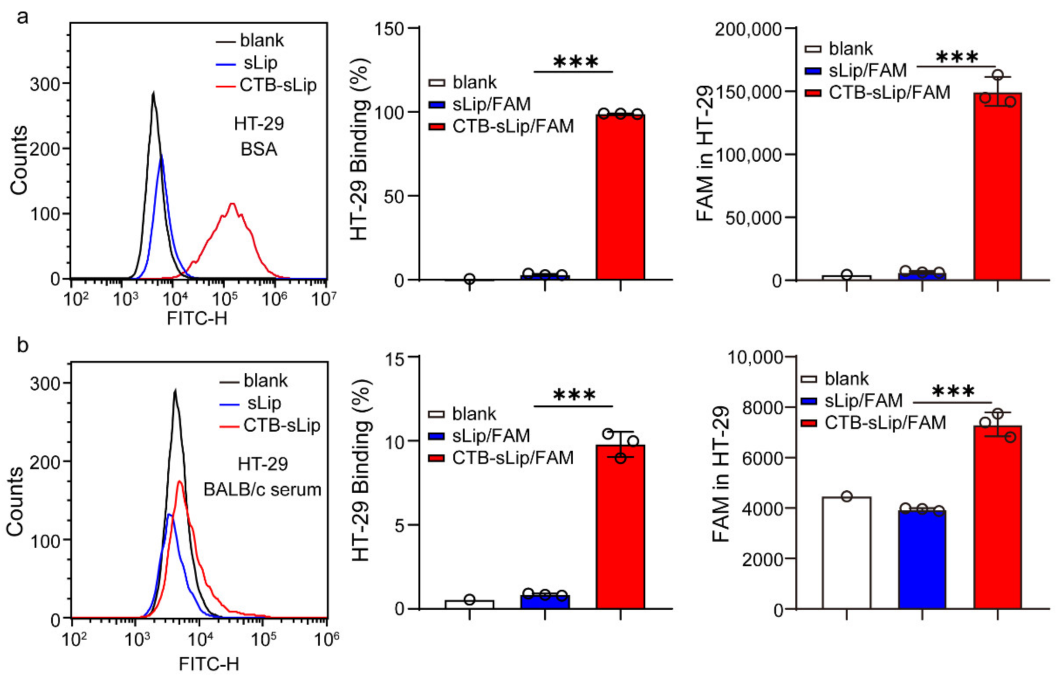

Supplementary Figure S4. The binding capability of CTB-sLip to HT-29 cells *in vitro*. FAM loaded liposomes (5 µM FAM) were incubated with HT-29 cells in 0.1 % BSA (a) and 10 % BALB/c serum (b) at 4 ℃ for 2 h (n =3). The blank is the same when comparing the difference between BSA and BALB/c serum. Data are means ± SDs. *** p < 0.001 by one-way ANOVA analyses with Prism.8.0.1.

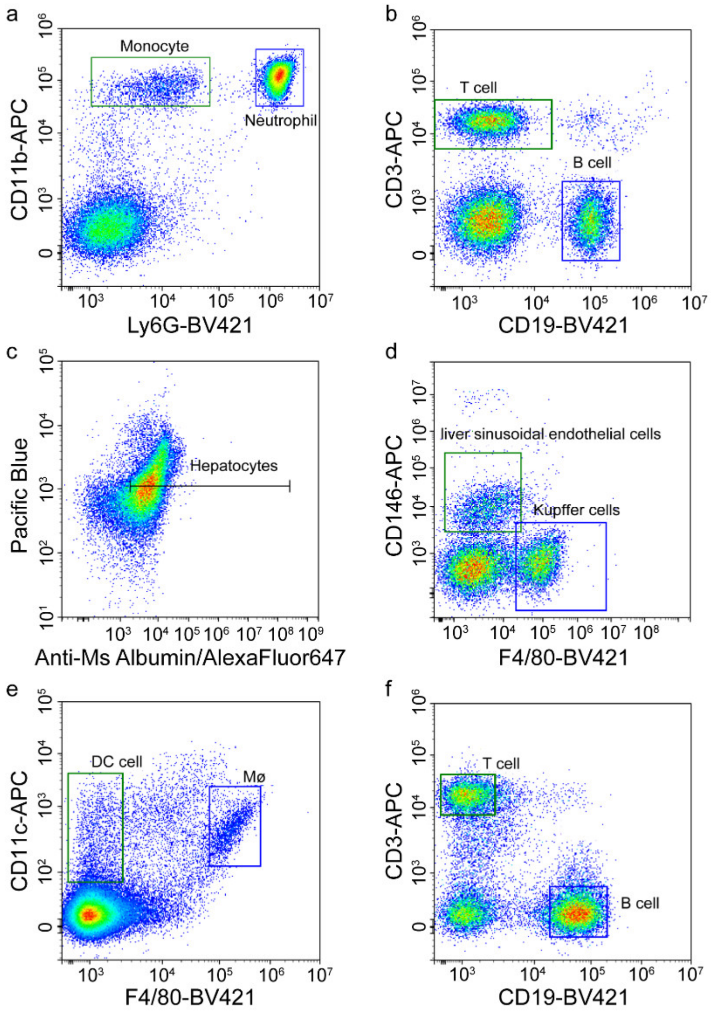

Supplementary Figure S5. Characterization of antibody strained cells by flow-cytometry. (a-b) The strained cells of peripheral blood cells (monocyte, neutrophil, T cells and B cells). (c-d) The strained cells of liver cells (hepatocytes, LSEC and Kupffer cells). (e-f) The strained cells of splenic cells (DC cells, Mϕ, T cells and B cells).

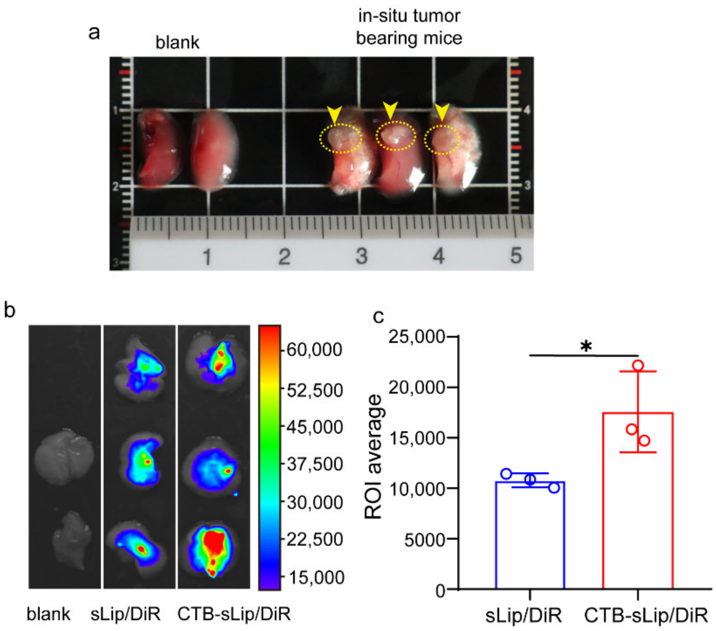

Supplementary Figure S6. Targeting capability of CTB-sLip to lung metastasis of colorectal cancer *in vivo*. (a) Male BALB/c nude mice planted with HCT-116 cells in left lungs. Tumor tissues are indicated by yellow arrows. (b) Xenografted mice were injected with sLip/DiR or CTB-sLip/DiR (50 mg lipid per kg of mice weight), and the whole left lung with implanted colorectal cancer cells was dissected for *in situ* imaging. (c) The fluorescence DiR in Region of Interest (ROI) was calculated from (b). Data are means ± SDs ($n = 3$). * $p < 0.05$ by student t-tests.
